# Surveillance of SARS-CoV-2 variants in Henan, China from 2023 to 2024

**DOI:** 10.3389/fcimb.2025.1511114

**Published:** 2025-02-11

**Authors:** Yun Song, Bicong Wu, Hongxia Ma, Yafei Li, Su Yan, Jingjing Pan, Haifeng Wang, Ying Ye, Xueyong Huang, Wanshen Guo

**Affiliations:** Henan Province Center for Disease Control and Prevention, Infectious Disease Prevention and Control Institute, Henan Provincial Key Laboratory of Infectious Disease Pathogens, Zhengzhou, Henan, China

**Keywords:** SARS-CoV-2, BA.5.2, XBB, BA.2.86, clinical characteristics

## Abstract

**Objective:**

In January 2023, China implemented the “Class B Management” policy, marking a new phase in COVID-19 control. As new SARS-CoV-2 variants continue to emerge, some have shown significant immune evasion, posing challenges to epidemic control efforts. To manage the pandemic effectively, Henan Province launched a surveillance program for SARS-CoV-2 variants, systematically analyzing their clinical characteristics and epidemiological patterns.

**Methods:**

This study collected genomic sequence data from 5,965 COVID-19 cases between January 1, 2023, and March 17, 2024, using the Henan Province SARS-CoV-2 variant surveillance system. Genome sequence analysis was performed with CLC Genomics Workbench, and genotyping and sequence alignment were carried out using the Nextclade platform. The clinical severity of different variants was assessed in relation to patient sex, age, clinical classification, and vaccination status.

**Results:**

Between Week 1 of 2023 and Week 11 of 2024, a total of 5,965 complete SARS-CoV-2 genome sequences were obtained, including 3,004 male (50.36%) and 2,961 female (49.64%) cases. The majority of cases were mild (5,451 cases, 91.38%), followed by moderate (311 cases, 5.21%) and severe or critical cases (203 cases, 3.4%). The predominant variants included BA.5.2, XBB, and BA.2.86. BA.5.2 was dominant until April 2023, after which it was gradually replaced by XBB. From December 2023, BA.2.86 began to increase and became the predominant variant by January 2024. The XBB variant exhibited a significantly lower rate of severe cases, with most infections being mild (*P* < 0.05). Male patients, the elderly, and certain variants (e.g., BA.5.2) were associated with more severe outcomes, while XBB and BA.2.86 showed lower pathogenicity, with a marked reduction in severe and fatal cases (*P* < 0.05).

**Conclusion:**

As SARS-CoV-2 variants evolve, the incidence of severe cases has progressively decreased. Both XBB and BA.2.86 variants exhibit lower pathogenicity. This study provides vital scientific evidence on the epidemiological features, clinical manifestations, and control strategies of SARS-CoV-2 variants. It underscores the importance of continuous viral surveillance and genomic sequencing to guide public health decision-making.

## 1 Introduction

On January 8, 2023, China adopted a “Class B management” approach for COVID-19, signaling the start of a new phase in pandemic control (NHC, 2022). On May 5 of the same year, the World Health Organization (WHO) declared that the COVID-19 pandemic no longer met the criteria for a “Public Health Emergency of International Concern” (PHEIC). However, SARS-CoV-2 continues to spread globally, with new variants emerging regularly. Monitoring data reveals the frequent appearance of these variants, many of which exhibit strong immune evasion properties. This has led to a gradual reduction in antibody protection, triggering new waves of infections caused by different variants. Consequently, the WHO has recommended that countries maintain surveillance of SARS-CoV-2 and continue genomic sequencing to assess risks and prepare for potential future outbreaks (WHO, 2024).

At the end of 2022, Henan Province launched a province-wide monitoring program for SARS-CoV-2 variants. By sequencing the genomes of representative COVID-19 samples, the program tracked the composition and distribution of circulating variants, facilitating the timely identification of new strains and the evaluation of their transmissibility, pathogenicity, and immune evasion properties. This study consolidates monitoring data from Week 1 of 2023 to Week 11 of 2024 (January 1, 2023, to March 17, 2024), encompassing 63 weeks of surveillance. It offers a comprehensive analysis of the clinical features and epidemiological trends of various SARS-CoV-2 variants.

## 2 Methods

### 2.1 Data source

This study analyzes whole-genome sequencing data from 5,965 COVID-19 cases reported by prefecture-level cities in Henan Province, as recorded in the SARS-CoV-2 variant monitoring information system, covering the period from the 1st week of 2023 to the 11th week of 2024 (January 1, 2023, to March 17, 2024). Ethical approval for this study was granted by the Medical Ethics Committee of the Henan Provincial Center for Disease Control and Prevention (approval number: 2020-KY-010).

### 2.2 Analytical method

This study gathered data on the gender, age, clinical classification, and vaccination history of COVID-19 patients from the SARS-CoV-2 variant monitoring information system in Henan Province. The clinical classification of these patients was based on the “Diagnosis and Treatment Protocol for COVID-19 (Trial Version 10)” issued by the National Health Commission of China, ensuring adherence to the most current clinical guidelines [The National Health Commission of the People’s Republic of China, 2023; NHC. (2023)].

### 2.3 Sequence alignment and analysis

The raw sequencing data were processed and assembled using CLC Genomics Workbench (Version 20.0, Qiagen, Germany). Single nucleotide polymorphisms (SNPs) were identified by aligning the assembled sequences with the Wuhan-Hu-1 genome (GenBank: MN908947) as the reference. Viral genome typing and multiple sequence alignments were conducted using the online platform Nextclade (https://clades.nextstrain.org/), which facilitated efficient classification and comparison of viral strains.

### 2.4 Statistical analysis

Data were organized and analyzed using Excel and SPSS 22.0. Categorical data are expressed as frequencies and percentages (%). For continuous data, normally distributed variables are presented as mean ± standard deviation ( $\bar{\text{x}}$ ± s), while skewed data are represented by the median [P50 (P25, P75)]. The χ² test was used to assess differences in clinical symptom distributions among different variants, and one-way analysis of variance (ANOVA) was employed to compare the rates of severe cases during the circulation of various variants. Statistical significance was set at *P* < 0.05. Graphs and charts were created using Origin software.

## 3 Results

### 3.1 Analysis and comparison of the epidemiological features of COVID-19 cases

**Figure 1:**
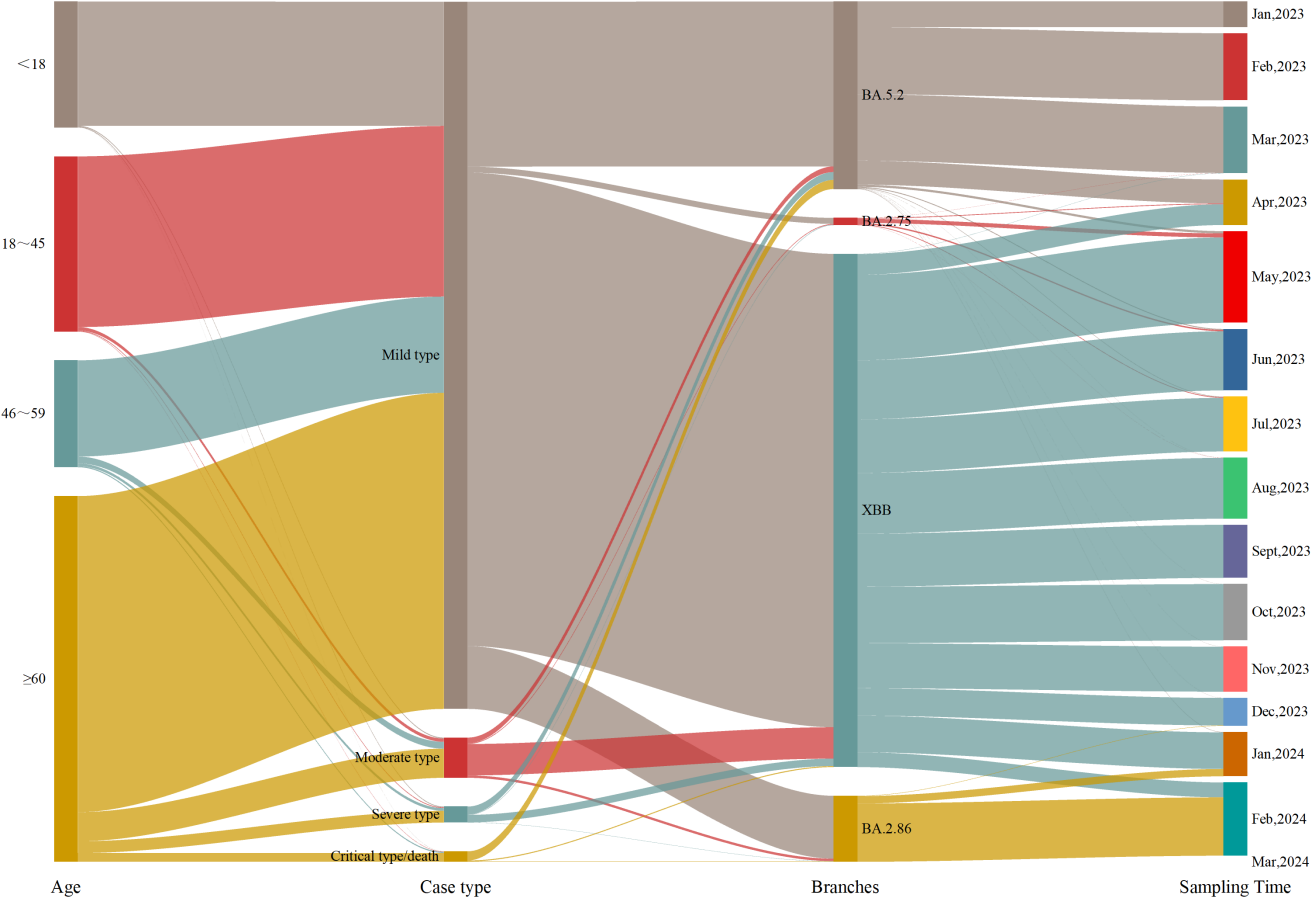
Epidemiological information of the cases.

From Week 1 of 2023 to Week 11 of 2024, a total of 5965 COVID-19 genomic sequences were obtained. Among these, 3004 cases (50.36%) were male and 2961 cases (49.64%) were female, resulting in a male-to-female ratio of 1.02:1. The ages of the cases ranged from 7 days to 117 years, with a median age of 57.44 years(interquartile range: 28.20-74.21 years). In terms of age distribution, there were 970 cases (16.26%) were under 18 years, 1342 cases (22.50%) were between 18 and 45 years, 833 cases (13.96%) were between 46 and 59 years, and 2820 cases (47.28%) were 60 years or older. Regarding clinical classification, 5451 cases (91.38%) were mild, 311 cases (5.21%) were moderate, 123 cases (2.06%) were severe, 80 cases (1.34%) were critical, and 1 case (0.02%) resulted in death. Regarding vaccination status, 5043 cases (84.54%) were vaccinated, 680 cases (11.40%) were unvaccinated, and 242 cases (4.06%) had missing immunization history. With respect to prior COVID-19 infection, 2439 cases (40.89%) had been previously infected, 3277 cases (54.94%) had no prior infection, and 249 cases (4.17%) had missing data on prior infection. In terms of variant distribution, 1435 cases (24.06%) were classified as the BA.5.2 lineage, 3929 cases (65.87%) as the XBB lineage, 56 cases (0.94%) as the BA.2.75 lineage, 541 cases (9.07%) as the BA.2.86 lineage, and 4 cases (0.07%) as other lineages (**Figure 1**).

To better understand the factors influencing the clinical severity of COVID-19, this study examined the distribution of disease severity across genders, age and viral strains. Regarding gender, the proportions of severe cases (2.60% vs. 1.52%) and critical/death cases (1.76% vs. 0.91%) were higher in males than in females. Chi-square test results revealed a significant association between gender and disease severity (*p* < 0.05), indicating that males are at greater risk of developing severe and critical cases. Analysis by age group showed that mild cases predominated across all age groups, but the proportion of severe and critical/death cases increased with age. Among patients aged 60 and older, 86.42% had mild cases, while 3.19% had severe cases and 2.38% had critical/death cases. Kruskal-Wallis test results indicated a significant difference in disease severity between age groups (*p* < 0.05), suggesting that older patients are more likely to progress to severe and critical conditions. Regarding viral strains, the BA.5.2 lineage exhibited higher proportions of severe and critical/death cases (4.18% and 4.88%, respectively). In contrast, XBB, BA.2.86, and other strains showed significantly lower proportions of severe and critical/death cases, with BA.2.86 showing only 0.37%. The BA.2.75 lineage, however, was associated with higher proportions of mild (10.71%) and severe (7.14%) cases but no critical/death cases. Chi-square test results indicated significant differences in disease severity across viral strains (*p* < 0.05), with the BA.5.2 lineage being associated with a higher risk of severe and critical cases, while BA.2.75 was more strongly associated with mild cases. In conclusion, while mild cases predominated, severe and critical cases were more common among older individuals and those infected with specific viral strains (**Table 1**).

**Table 1:** Impact of gender, age, and viral strains on clinical symptoms in COVID-19 cases.

		Casetype			
		Mildtype	Moderatetype	Severetype	Criticaltype/death
		n (%)	n (%)	n (%)	n (%)
Gender					
	Male	2673 (88.98)	200 (6.66)	78 (2.6)	53 (1.76)
	Female	2778 (93.82)	111 (3.75)	45 (1.52)	27 (0.91)
Age					
	<18	956 (98.56)	7 (0.72)	6 (0.62)	1 (0.10)
	18˜45	1307 (97.39)	26 (1.94)	6 (0.45)	3 (0.22)
	46˜59	751 (90.16)	52 (6.24)	21 (2.52)	9 (1.08)
	≥60	2437 (86.42)	226 (8.01)	90 (3.19)	67 (2.38)
Lineages					
	BA.5.2	1262 (87.94)	43 (3.00)	60 (4.18)	70 (4.88)
	XBB	3624 (92.24)	240 (6.11)	57 (1.45)	8 (0.20)
	BA.2.86	515 (95.19)	22 (4.07)	2 (0.37)	2 (0.37)
	BA.2.75	46 (82.14)	6 (10.71)	4 (7.14)	0 (0)
	Others	4 (100)	0 (0)	0 (0)	0 (0)
Total n (%)		5451 (91.38)	311 (5.21)	123 (2.06)	80 (1.34)

### 3.2 Monitoring results of SARS-CoV-2 variants

**Figure 2:**
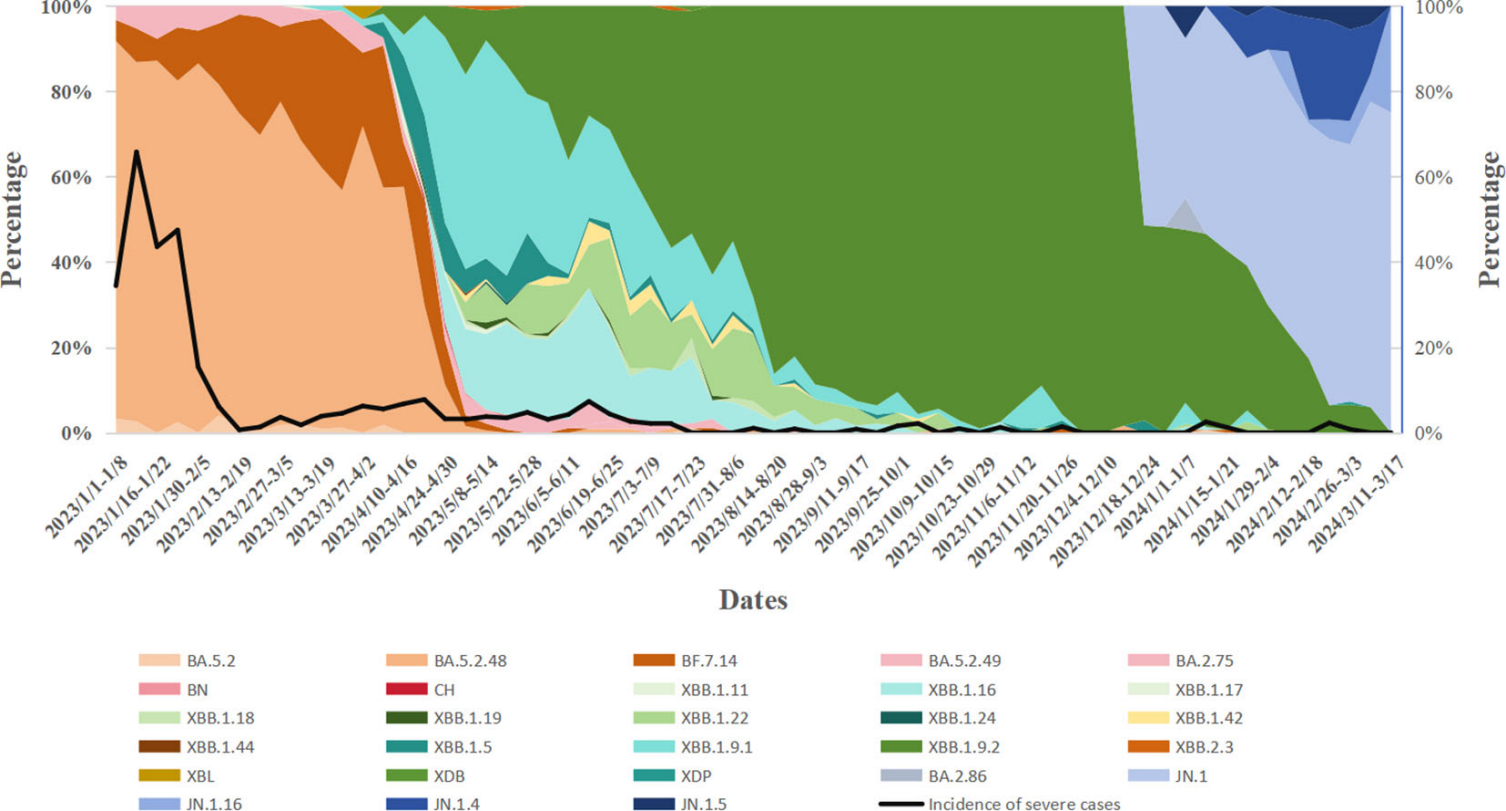
Temporal dynamics of SARS-CoV-2 variant prevalence and their correlation with variations in disease severity rates.

During Week 1 (January 1) and Week 16 (April 23) of 2023,the BA.5.2 lineage was the predominant variant, accounting for over 50% of cases each week. However, in Week 17 (April 24-30), its proportion dropped to 21.77%, and from Week 18 (May 1-7) onwards, the proportion of BA.5.2 detected each week remained below 5%. The XBB lineage was first detected on March 6, 2023, and its proportion gradually increased. From Week 17 of 2023 (April 24-30; 74.19%) to Week 4 of 2024 (January 22-28; 64.29%), XBB gradually replaced BA.5.2 as the dominant variant. However, from Week 8 (February 19-25, 2024) onward, XBB’s proportion declined to below 10%. The BA.2.75 lineage was first detected on March 17, 2023, and its proportion slowly increased, always remaining below 5%. It was not detected again after August 24, 2023. The BA.2.86 lineage emerged on December 19, 2023,and its share gradually increased, surpassing XBB to become the dominant variant by Week 5 of 2024 (January 29-February 4; 57.14%). As of Week 11 of 2024, BA.2.86 remains the predominant variant in Henan Province. During this period, Henan Province detected two cases of the XBL recombinant variant on March 28 and 30, 2023, one case of the XDB recombinant variant on July 18, 2023, and one case of the XDP recombinant variant on February 27, 2024 (**Figure 2**).

From January 1, 2023, to March 17, 2024, the prevalence of various variants, including BA.5.2, XBB, and the BA.2.86 evolutionary branch, fluctuated over time. As these variants evolved, a noticeable decline in case severity was observed. In early 2023, severity rates during certain periods approached 30%-40%. By September 2023, the severity rate had significantly decreased, with most weeks reporting rates below 10%. A one-way analysis of variance (ANOVA) comparing severity rates across different variants revealed significant differences (*P* < 0.05). Specifically, earlier variants (e.g., the BA.5.2 series) were associated with higher severity rates, whereas later variants (e.g., XBB and BA.2.86) were linked to lower severity rates. Notably, a sharp decline in severity was observed during the transition from BA.5.2 to the XBB series (**Table 2**).

**Table 2:** Clinical symptom distribution across different BA.5.2 sublineages.

		Casetype			
		Mildtype	Moderatetype	Severetype	Criticaltype/death
		n (%)	n (%)	n (%)	n (%)
Lineages					
	BA.5.2.48	883 (87.17)	25 (5)	47 (4.64)	58 (5.73)
	BA.5.2.49	35 (66.04)	8 (2.47)	3 (5.66)	7 (13.21)
	BF.7.14	328 (93.98)	9 (15.09)	7 (2.01)	5 (1.43)
	Others	16 (80)	1 (2.58)	3 (15)	0 (0)
Total n (%)		1262 (87.94)	43 (3)	60 (4.18)	70 (4.88)

#### 3.2.1 Analysis of the BA.5.2 variants

**Figure 3:**
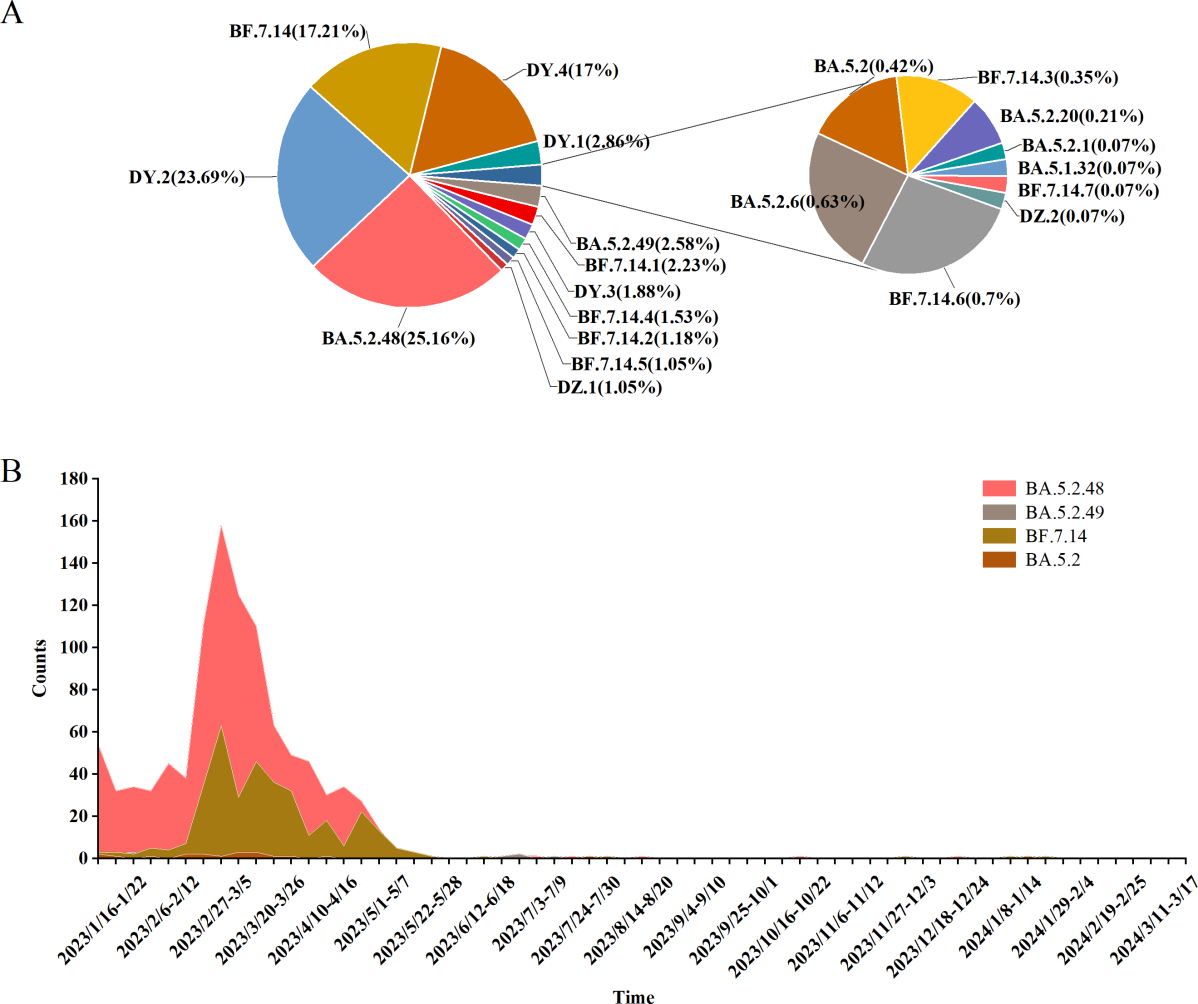
Lineage Distribution of BA.5.2 (Including BA.5.2.48, BF.7.14, BA.5.2.49, and Other Sublineages of BA.5.2). **(A)** Prevalence of the BA.5.2 Variant in Henan Province, China, from January 1, 2023, to March 17, 2024. **(B)** Distribution of BA.5.2 Sublineages Over Time.

In this study, 5,965 COVID-19 genomic sequences were analyzed, with the BA.5.2 lineage representing 24.06% (1,435/5,965) of the total, comprising 20 distinct sublineages. The three most prevalent sublineages were BA.5.2.48 (361 cases, 25.16%), DY.2 (340 cases, 23.69%), and BF.7.14 (247 cases, 17.21%). Further breakdown of the BA.5.2 lineage revealed that BA.5.2.48 (5 sublineages) accounted for 1,013 cases (70.59%), BF.7.14 (7 sublineages) accounted for 349 cases (24.32%), BA.5.2.49 (3 sublineages) accounted for 53 cases (3.69%), and the other BA.5.2 lineage (5 sublineages) accounted for only 20 cases (1.39%) (**Figure 3A**).

From Week 1 to Week 16 of 2023 (January 1 to April 23), the BA.5.2 lineage remained the dominant circulating variant, consistently accounting for over 50% of cases each week. Among its sublineages, BA.5.2.48 was most prevalent in Week 1, comprising 88.52% of cases. However, its proportion gradually declined to 57.63% by Week 15 and further dropped to 30% by Week 16. In contrast, the proportion of BF.7.14 steadily increased, starting at 4.92% in Week 1 and rising to 24.44% by Week 16, nearing the proportion of BA.5.2.48 in the same week. Meanwhile, BA.5.2.49 showed a relatively stable trend, with its proportion fluctuating at low levels throughout the 16-week period, consistently remaining below 10%. In conclusion, the BA.5.2 lineage, particularly the BA.5.2.48 sublineage, dominated the epidemic during this period, although other variants also co-circulated (**Figure 3B**).

An analysis of clinical data from 1,435 cases of the BA.5.2 lineage of SARS-CoV-2 revealed that the majority of infections were classified as mild (1,262 cases, 87.94%), followed by severe (60 cases, 4.18%), critical/death (70 cases, 4.88%), and moderate (43 cases, 3%). This distribution suggests that the majority of BA.5.2 infections are mild, with relatively few severe or fatal outcomes. A chi-square test was performed to assess the differences in clinical manifestations (mild, moderate, severe, critical/death) across subvariants, and the results indicated significant statistical differences in the distribution of clinical types among the variants (*P*<0.05). Among the BA.5.2 subvariants, BA.5.2.49 exhibited a notably higher proportion of critical/death cases (13.21%), significantly higher than those of other variants. This suggests that BA.5.2.49 may possess enhanced pathogenicity, potentially leading to a higher incidence of severe and fatal outcomes. In contrast, BF.7.14 was predominantly associated with mild cases (93.98%), with the majority of infected individuals exhibiting mild or asymptomatic symptoms. This indicates that BF.7.14 has relatively lower pathogenicity and a reduced risk of severe illness and death. BA.5.2.48 also exhibited some pathogenic risk, with 5.73% of cases classified as critical/death. Although this proportion is lower than that of BA.5.2.49 (13.21%), it remains higher than that of BF.7.14.

#### 3.2.2 Analysis of the XBB variants

**Figure 4:**
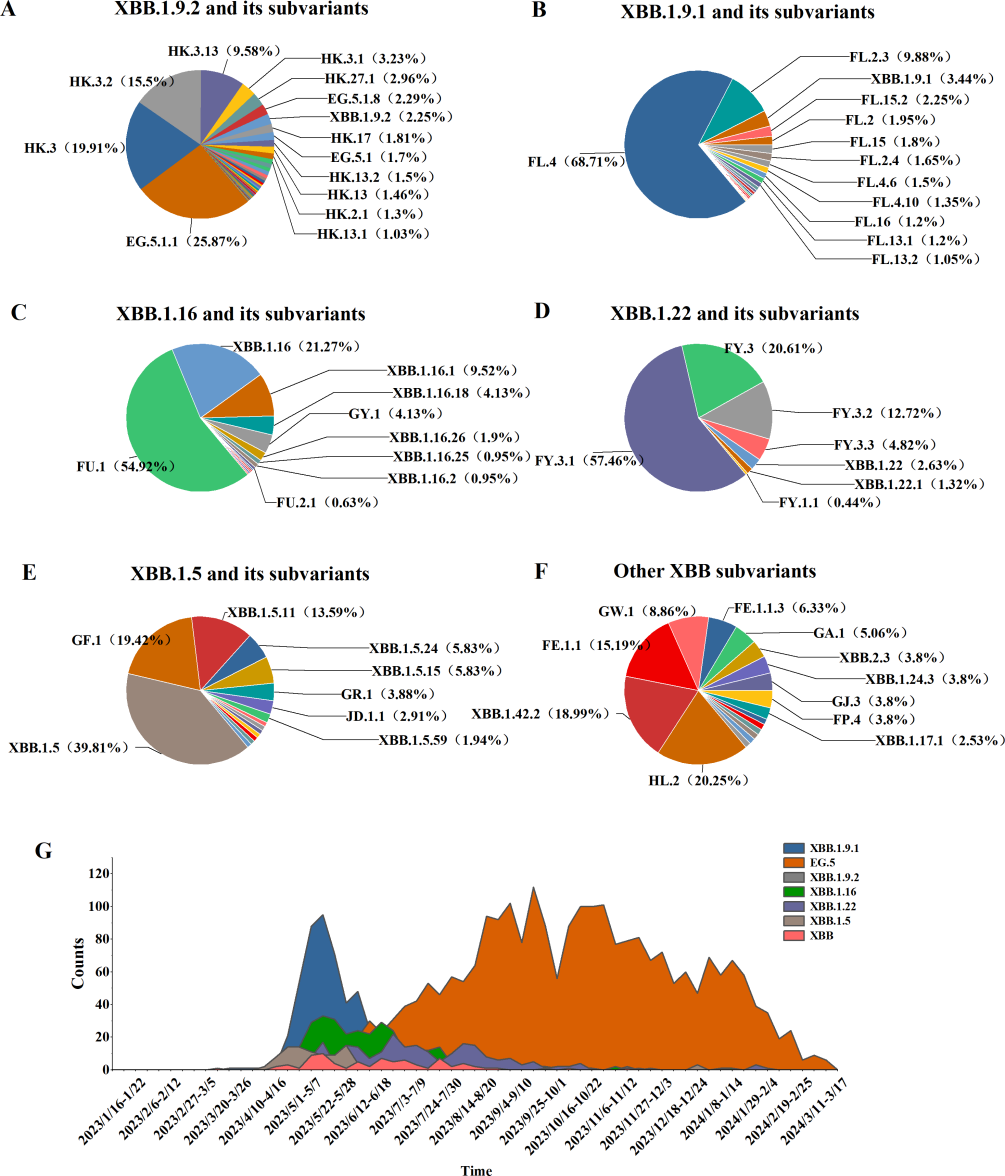
Distribution of XBB lineages, including XBB.1.16, XBB.1.22, XBB.1.5, XBB.1.9.1, XBB.1.9.2, and other sub-lineages of XBB. **(A–F)** Prevalence of the XBB variant in Henan Province from January 1, 2023, to March 17, 2024 **(G)**.

Among 5,965 SARS-CoV-2 genomic sequences from COVID-19 cases, the XBB lineage accounted for 65.87% (3,929/5,956), encompassing 128 distinct evolutionary branches. The top three branches by prevalence were EG.5.1.1 (656 cases, 16.7%), HK.3 (505 cases, 12.85%), and FL.4 (459 cases, 11.68%). Further classification of the XBB lineage revealed the following distribution: XBB.1.9.2 and its subvariants (including 53 branches) accounted for 2,536 cases (64.55%), XBB.1.9.1 and its subvariants (including 24 branches) accounted for 688 cases (17%), XBB.1.16 and its subvariants (including 14 branches) accounted for 315 cases (8.02%), XBB.1.22 and its subvariants (including 7 branches) accounted for 228 cases (5.8%), XBB.1.5 and its subvariants (including 15 branches) accounted for 103 cases (2.62%), and other XBB subvariants (including 15 branches) accounted for 79 cases (2.01%) (**Figures 4A–F**).

From week 17 of 2023 (April 24–30) to week 4 of 2024 (January 22–28), the XBB lineage was the dominant circulating variant. Between weeks 17 and 27, several XBB subvariants, including XBB.1.9.1, XBB.1.9.2, XBB.1.16, XBB.1.22, and XBB.1.5, co-circulated. However, from week 28 to week 10 of 2024 (July 10, 2023, to March 10, 2024), the EG.5 lineage emerged as the predominant XBB subvariant, marking a shift in its dominance during the later phase of circulation (**Figure 4G**).

Among 3,929 genomic sequences of the XBB evolutionary branch of SARS-CoV-2, mild cases accounted for 92.24% (3,624 cases), moderate cases for 6.11% (240 cases), severe cases for 1.45% (57 cases), and critical or fatal cases for 0.2% (8 cases), with mild cases constituting the majority. Clinical classification analysis revealed significant differences in the distribution of clinical symptoms across different XBB subbranches (*P* < 0.05). Specifically, the XBB.1.5 subbranch had the highest proportion of mild cases (95.15%) and no severe or critical cases, suggesting lower pathogenicity, with the majority of cases presenting as mild. The XBB.1.9.2 subbranch also showed a high proportion of mild cases (93.38%), but compared to XBB.1.5, it exhibited a slight increase in severe and critical cases (0.67%), indicating marginally higher pathogenicity, although severe cases remained relatively rare. The XBB.1.9.1 and XBB.1.16 subbranches had mild case proportions of 89.97% and 91.11%, respectively. However, compared to XBB.1.5, both subbranches showed a notable increase in severe and critical cases (3.74% and 3.81%, respectively), suggesting moderately higher pathogenicity. The XBB.1.22 subbranch, while having a higher proportion of moderate cases (8.77%) and 88.16% mild cases, exhibited a low incidence of severe cases (3.07%) and no critical cases, suggesting relatively stronger pathogenicity, but a lower risk of severe disease (**Table 3**).

**Table 3:** Clinical symptom distribution across different XBB sublineages.

		Casetype			
		Mildtype	Moderatetype	Severetype	Criticaltype/death
		n (%)	n (%)	n (%)	n (%)
Lineages					
	XBB.1.9.1	601 (89.97)	42 (6.29)	22 (3.29)	3 (0.45)
	XBB.1.9.2	2368 (93.38)	151 (5.95)	13 (0.51)	4 (0.16)
	XBB.1.16	287 (91.11)	16 (5.08)	11 (3.49)	1 (0.32)
	XBB.1.22	201 (88.16)	20 (8.77)	7 (3.07)	0 (0)
	XBB.1.5	98 (95.15)	5 (4.85)	0 (0)	0 (0)
	Others	69 (87.34)	6 (7.59)	4 (5.06)	0 (0)
Total n (%)		3624 (92.24)	240 (6.11)	57 (1.45)	8 (0.2)

#### 3.2.3 Analysis of the BA.2.86 variant

**Figure 5:**
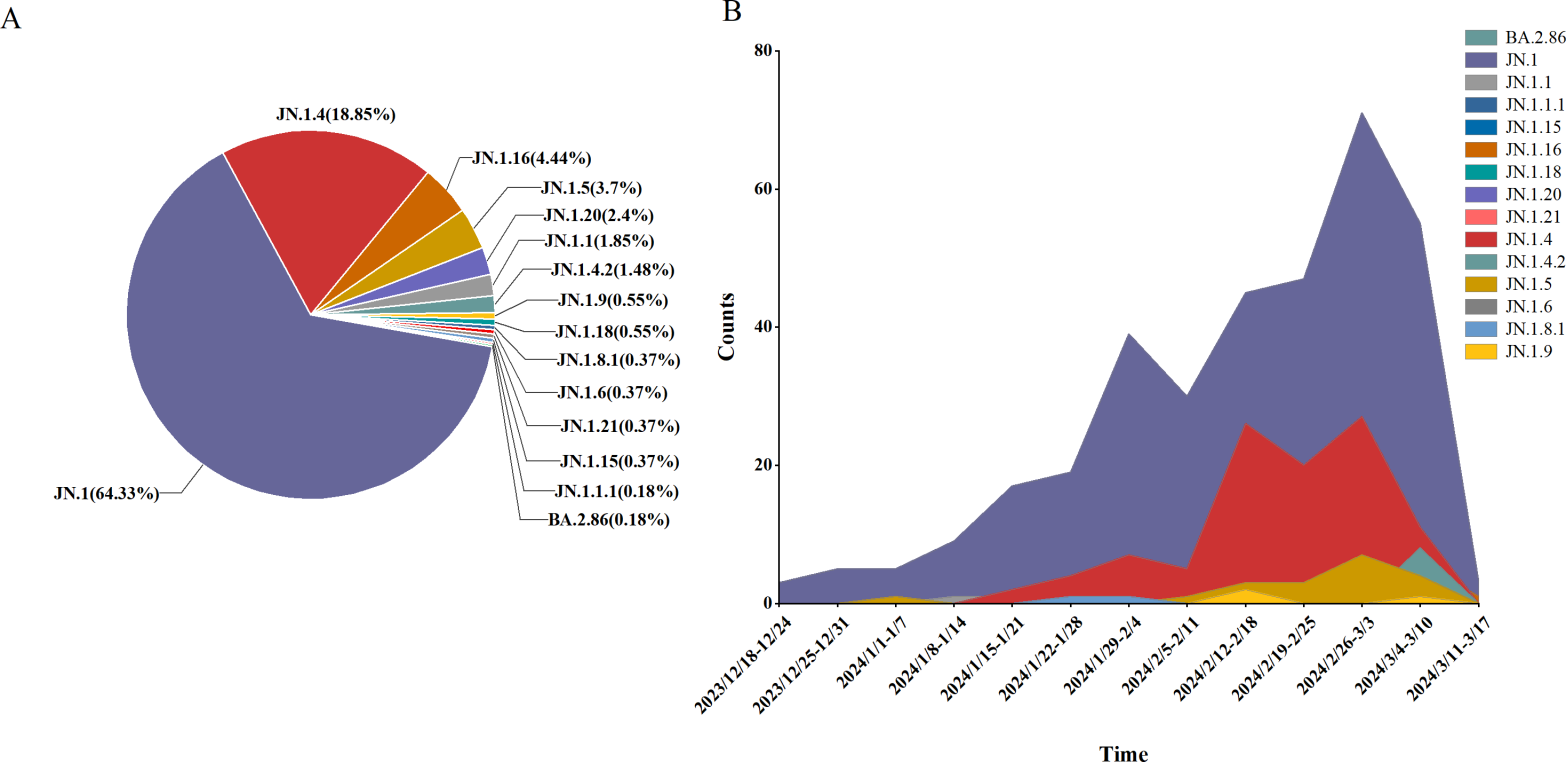
Lineage distribution of BA.2.86, including JN.1, JN.1.16, JN.1.4, JN.1.5, and other sub-lineages of BA.2.86. **(A)** The prevalence of BA.2.86 variant in Henan Province from January 1, 2023 to March 17, 2024 **(B)**.

An analysis of 541 SARS-CoV-2 genome sequences from COVID-19 cases found that the BA.2.86 lineage accounted for 9.07% (541/5956), comprising 15 distinct sublineages. The most prevalent sublineages were JN.1 (348 cases, 64.33%), JN.1.4 (102 cases, 18.85%), and JN.1.16 (24 cases, 4.44%). Between weeks 5 and 11 of 2024 (January 29 to March 17), the BA.2.86 lineage emerged as the dominant circulating variant. During this period, JN.1 was the primary circulating sublineage, consistently accounting for over 50% of cases each week, alongside co-circulating sublineages such as JN.1.4, JN.1.5, and JN.1.16 (**Figure 5**).

Clinical classification of the 541 BA.2.86 cases showed that mild cases comprised 95.19% (515 cases), moderate cases 4.07% (22 cases), severe cases 0.37% (2 cases), and critical/death cases 0.37% (2 cases). Chi-square analysis revealed significant differences in the distribution of clinical classifications (mild, moderate, severe, and critical/death) across sublineages (*P* < 0.05). Specifically, all cases of the JN.1.16 and JN.1.5 sublineages were mild, with no moderate, severe, or critical cases, suggesting lower pathogenicity and predominantly mild symptoms. In contrast, the JN.1 and JN.1.4 sublineages had a higher proportion of moderate cases, particularly JN.1 (4.60%), indicating slightly higher pathogenicity, though mild cases still predominated. Despite slight variations in severe case proportions across sublineages, chi-square analysis revealed no significant differences in the rates of severe illness (*P* > 0.05) (**Table 4**).

**Table 4:** Clinical symptom distribution across different BA.2.86 sublineages.

		Casetype			
		Mildtype	Moderatetype	Severetype	Criticaltype/death
		n(%)	n(%)	n(%)	n(%)
Lineages					
	JN.1	329(94.54)	16(4.6)	2(0.57)	1(0.29)
	JN.1.16	24(100)	0(0)	0(0)	0(0)
	JN.1.4	97(95.10)	4(3.92)	0(0)	1(0.98)
	JN.1.5	20(100)	0(0)	0(0)	0(0)
	Others	45(95.74)	2(4.26)	0(0)	0(0)
Total n(%)		515(95.19)	22(4.07)	2(0.37)	2(0.37)

## 4 Discussion

Since the identification of the SARS-CoV-2 virus in November 2019, it has undergone numerous mutations and spread globally. The Omicron variant, first detected in South Africa in November 2021, quickly disseminated worldwide, exhibiting more pronounced mutations than previous variants (Tong et al., 2022). Notably, several key changes in its spike protein have enabled the virus to evade neutralizing antibodies more effectively while significantly enhancing its transmissibility. As a result, breakthrough infections and reinfections have become widespread globally (Khan et al., 2024). Over time, the original Omicron variant BA.1 was gradually replaced by BA.2, which subsequently diverged into multiple subvariants, including BA.2.75, BA.4, BA.5, and BA.2.86. Among these, BA.5 and its subvariants have emerged as the dominant circulating strains, driving extensive infections and ongoing concerns (Verkhivker et al., 2023; Yajima et al., 2024). Currently, BA.2.86 is predominant in several countries, with sublineages such as JN.1, JN.1.16, and KP.2 representing new global evolutionary lineages (Satapathy et al., 2024). The concurrent circulation of multiple SARS-CoV-2 variants worldwide has garnered significant attention from both scientific and public health communities.

This study analyzes the monitoring data of SARS-CoV-2 variants in Henan Province from January 1, 2023, to March 17, 2024, with the aim of assessing the epidemiological trends, clinical manifestations, and their association with disease severity. The results reveal that, with the continuous evolution of variants, both transmission dynamics and clinical severity have significantly changed, particularly in terms of the decline in severe cases. Specifically, the study found that the prevalence of SARS-CoV-2 variants exhibited notable temporal fluctuations. BA.5.2 predominated from early 2023 until April; however, its prevalence sharply declined after Week 17, being rapidly replaced by the XBB lineage. The XBB lineage continued to dominate until Week 5 of 2024, after which the BA.2.86 lineage gradually emerged as the dominant strain. The epidemiological trends of different variants are likely linked to biological characteristics such as immune evasion and transmissibility. Notably, the replacement of BA.5.2 by the XBB and BA.2.86 variants suggests that these strains may have enhanced immune evasion or adaptability, which is closely associated with the ongoing challenges in global pandemic control.

The BA.5 variant first emerged in early January 2022 and rapidly spread worldwide. Compared to BA.2, BA.5 exhibits significant mutations in the receptor-binding domain (RBD), particularly at the L452R, F486V, and R493Q positions. These mutations enhance BA.5’s immune evasion capabilities, thereby increasing its transmissibility and contributing to the rise in breakthrough infections (Cocherie et al., 2022; Bai et al., 2024; Zhu et al., 2025). Additionally, the BF.7 subvariant, derived from BA.5, has accumulated further mutations, such as K444T and R346T, which may enhance its immune escape and transmissibility (Ma et al., 2024). Notably, BF.7.14 and BA.5.2.48 share identical mutations in the spike protein, with the exception of R346T and C1243F (Zhang et al., 2023). The conservation of these mutations suggests that the clinical manifestations of these two variants are quite similar. Our study found that both BA.5.2.48 and BF.7.14 primarily result in mild clinical symptoms, with no significant differences between the two. Related studies also suggest that BA.5.48 and BF.7.14 infections exhibit no notable differences in terms of age, gender, clinical manifestations, disease progression, or treatment outcomes (Huo et al., 2023; Lai et al., 2024). Moreover, BA.5.2.49 may demonstrate increased pathogenicity, potentially leading to more severe cases and deaths. Some studies suggest that BA.5.2.49 spreads more rapidly and is associated with higher hospitalization rates and mortality, which aligns with our findings (Huang et al., 2023).

XBB is a recombinant variant composed of the BJ.1 and BM.1.1.1 sublineages. BJ.1 originates from the BA.2.10 mutation, while BM.1.1.1 is derived from mutations in BA.2.75. First identified in India in August 2022, XBB subsequently gave rise to subvariants such as XBB.1.9, XBB.1.5, and XBB.1.16 (Kurhade et al., 2023). Compared to BA.2, XBB exhibits several additional mutations in the N-terminal domain (NTD) and receptor-binding domain (RBD), which may enhance its immune evasion capabilities (Wang et al., 2023b). XBB.1.5, in addition to carrying the mutations present in XBB, includes a critical mutation, F486P, which is also found in XBB.1.16. Furthermore, XBB.1.16 carries additional mutations-E180V in the NTD and T478R in the RBD—that may further strengthen its immune escape properties (Peng et al., 2024). In our study, the transition from BA.5.2 to the XBB lineage was associated with a marked reduction in disease severity. Notably, among the XBB subvariants, XBB.1.5 exhibited a higher proportion of mild cases. Despite this, the multiple mutations in the RBD region of XBB and its subvariants enhance their immune escape capacity, potentially posing a sustained challenge for reinfections.

The BA.2.86 variant was first identified on October 22, 2022, and was quickly designated as a variant under monitoring (Yang et al., 2023). In comparison to BA.2.86, the JN.1 subvariant carries a single mutation (L455S) in the receptor-binding domain (RBD), which may alter the binding affinity between ACE2 and JN.1, thereby enhancing its immune evasion capabilities (Wang et al., 2023a). Research has shown that the incidence of severe disease and mortality in BA.2.86 cases is significantly lower than in those infected with pre-Omicron variants (Uriu et al., 2023). In this study, the incidence of severe and critical cases associated with BA.2.86 was significantly lower than that of the previously dominant XBB and BA.5.2 variants. Moreover, no significant differences in case severity were observed among the BA.2.86 sublineages, including JN.1, JN.1.16, JN.1.4, and JN.1.5, suggesting that BA.2.86 and its subvariants exhibit lower pathogenicity.

In addition, Age and sex are key determinants of the clinical severity of COVID-19. Our study indicates that men are more likely than women to develop severe or critical cases. Additionally, elderly individuals, particularly those aged 60 and above, make up a larger proportion of severe and critical cases, with the incidence increasing significantly in this age group. These findings suggest that older adults are more vulnerable to severe outcomes due to immune system decline and the presence of underlying comorbidities (Flook et al., 2021). Therefore, early intervention and vaccination, especially for high-risk groups such as the elderly, remain essential strategies in controlling the COVID-19 pandemic.

This study provides valuable insights into the dynamic evolution and epidemiological patterns of SARS-CoV-2 variants. While the findings enhance our understanding of COVID-19 variants in Henan Province, several limitations should be acknowledged. First, the analysis was based on a subset of viral genome sequences, which may introduce sampling bias. Second, due to the limited monitoring period, ongoing tracking and analysis of the evolving trends of various variants and their association with clinical symptoms are essential. Lastly, as new variants continue to emerge, continuous evaluation of viral characteristics and vaccine efficacy remains central to the global effort to combat the COVID-19 pandemic.

## 5 Conclusions

This study analyzes the surveillance data of SARS-CoV-2 variants in Henan Province, highlighting differences in epidemic trends, clinical manifestations, and pathogenicity across various variants. Over time, the severity of COVID-19 has significantly decreased; however, elderly individuals and those infected with specific variants remain at higher risk of severe clinical outcomes. Vaccination continues to be a crucial strategy for reducing the incidence of severe COVID-19 cases. Future research should focus on the emergence of new variants and the associated challenges, while further strengthening efforts in virus surveillance and vaccine development to adapt to the evolving nature of the pandemic.

## Data Availability

The datasets presented in this study can be found in online repositories. The names of the repository/repositories and accession number(s) can be found in the article/supplementary material.
